# The impact of HLA class I and EBV latency‐II antigen‐specific CD8^+^ T cells on the pathogenesis of EBV^+^ Hodgkin lymphoma

**DOI:** 10.1111/cei.12716

**Published:** 2015-11-13

**Authors:** K. Jones, L. Wockner, R. M. Brennan, C. Keane, P. K. Chattopadhyay, M. Roederer, D. A. Price, D. K. Cole, B. Hassan, K. Beck, D. Gottlieb, D. S. Ritchie, J. F. Seymour, F. Vari, P. Crooks, S. R. Burrows, M. K. Gandhi

**Affiliations:** ^1^ Blood Cancer Research, University of Queensland Diamantina Institute, Translational Research InstituteBrisbaneAustralia; ^2^ Clinical Immunohaematology LaboratoryQIMR Berghofer Medical Research InstituteBrisbaneAustralia; ^3^ Statistics Unit, QIMR Berghofer Medical Research InstituteBrisbaneAustralia; ^4^ Cellular Immunology LaboratoryQIMR Berghofer Medical Research InstituteBrisbaneAustralia; ^5^ Department of HaematologyPrincess Alexandra HospitalBrisbaneAustralia; ^6^ ImmunoTechnology Section, Vaccine Research CenterNational Institute of Allergy and Infectious Diseases, National Institutes of HealthBethesdaMDUSA; ^7^ Human Immunology Section, Vaccine Research CenterNational Institute of Allergy and Infectious Diseases, National Institutes of HealthBethesdaMDUSA; ^8^ Institute of Infection and Immunity, Cardiff University School of MedicineCardiffUK; ^9^ Tissue Engineering and Reparative DentistryCardiff University School of DentistryCardiffUK; ^10^ Blood and Marrow Transplant Service, Westmead HospitalSydneyAustralia; ^11^ Department of HaematologyPeter MacCallum Cancer CentreMelbourneAustralia; ^12^ University of MelbourneMelbourneAustralia

**Keywords:** classical Hodgkin lymphoma, Epstein–Barr virus, genetic associations, HLA class I, T cell immunity

## Abstract

In 40% of cases of classical Hodgkin lymphoma (cHL), Epstein–Barr virus (EBV) latency‐II antigens [EBV nuclear antigen 1 (EBNA1)/latent membrane protein (LMP)1/LMP2A] are present (EBV^+^cHL) in the malignant cells and antigen presentation is intact. Previous studies have shown consistently that HLA‐A*02 is protective in EBV^+^cHL, yet its role in disease pathogenesis is unknown. To explore the basis for this observation, gene expression was assessed in 33 cHL nodes. Interestingly, CD8 and LMP2A expression were correlated strongly and, for a given LMP2A level, CD8 was elevated markedly in HLA‐A*02^–^ *versus* HLA‐A*02^+^ EBV^+^cHL patients, suggesting that LMP2A‐specific CD8^+^ T cell anti‐tumoral immunity may be relatively ineffective in HLA‐A*02^–^ EBV^+^cHL. To ascertain the impact of HLA class I on EBV latency antigen‐specific immunodominance, we used a stepwise functional T cell approach. In newly diagnosed EBV^+^cHL, the magnitude of *ex‐vivo* LMP1/2A‐specific CD8^+^ T cell responses was elevated in HLA‐A*02^+^ patients. Furthermore, in a controlled *in‐vitro* assay, LMP2A‐specific CD8^+^ T cells from healthy HLA‐A*02 heterozygotes expanded to a greater extent with HLA‐A*02‐restricted compared to non‐HLA‐A*02‐restricted cell lines. In an extensive analysis of HLA class I‐restricted immunity, immunodominant EBNA3A/3B/3C‐specific CD8^+^ T cell responses were stimulated by numerous HLA class I molecules, whereas the subdominant LMP1/2A‐specific responses were confined largely to HLA‐A*02. Our results demonstrate that HLA‐A*02 mediates a modest, but none the less stronger, EBV‐specific CD8^+^ T cell response than non‐HLA‐A*02 alleles, an effect confined to EBV latency‐II antigens. Thus, the protective effect of HLA‐A*02 against EBV^+^cHL is not a surrogate association, but reflects the impact of HLA class I on EBV latency‐II antigen‐specific CD8^+^ T cell hierarchies.

## Introduction

Epstein–Barr virus (EBV) is a ubiquitous, persistent B cell‐trophic virus that typically establishes a benign infection of minimal clinical consequence. However, in a minority of hosts, EBV is associated with particular malignancies. In these conditions, EBV resides within the malignant cell in a restricted state of latent antigen expression. The frequency of association and expression of latent proteins is distinctive to the type of cancer. In classical Hodgkin lymphoma (cHL), approximately 40% of cases are associated with EBV (EBV^+^cHL), and virus expression in the Hodgkin–Reed–Sternberg (HRS) cells is limited to the EBV nuclear antigen 1 (EBNA1) and latent membrane proteins (LMP1 and LMP2)^1^, ^2^, ^3^. These are collectively termed EBV latency‐II antigens, a pattern of expression also observed in undifferentiated nasopharyngeal carcinoma. EBV^+^cHL is characterized by intact human leucocyte antigen (HLA) class I antigen processing and presentation 4, 5, 6, 7, enabling successful treatment of relapsed EBV^+^cHL by adoptive immunotherapy targeting EBV latency‐II antigens 8, 9, 10, 11.

A well‐established hierarchy exists among CD8^+^ T cell responses that target EBV latency antigens. In particular, EBV latency‐III antigens EBNA3A/3B/3C are immunodominant, whereas the EBV latency‐II antigens (EBNA1/LMP1/LMP2A) are subdominant and more challenging to detect without *in‐vitro* expansion 12, 13. However, the impact of HLA class I on EBV latency antigen‐specific CD8^+^ T cell immunity has not been determined systematically. Interestingly, large epidemiological and genomewide association studies have consistently reported differential HLA class I susceptibility to EBV^+^cHL (Supporting information, Table S1) 14, 15, 16, 17. In western European populations, HLA‐A*01 and HLA‐B*37 are associated with increased susceptibility to EBV^+^cHL, while HLA‐A*02 is associated with protection 15, 16, 17. By contrast, the HLA‐A*02 subtype HLA‐A*0207, which presents HLA‐A*0201‐restricted LMP2A‐derived peptides poorly 18, is over‐represented in northern Chinese EBV^+^cHL patients 19. Non‐HLA‐linked genetic susceptibility loci have also been identified for cHL, as has a single nucleotide polymorphism (SNP) found in association with an HLA class II locus. However, these associations were not specific for EBV^+^cHL 14, 20, 21.

The aim of this study was to understand the role of HLA class I in the pathogenesis of EBV^+^cHL. The presentation of viral peptide determinants by HLA‐A*02 and non‐HLA‐A*02 molecules provides a potential mechanistic link between EBV latency‐II‐specific CD8^+^ T cell immunity and the described genetic associations with EBV^+^cHL. However, there are many genes with diverse functions in close proximity to HLA class I. Therefore, such associations may simply reflect linkage disequilibrium between HLA class I and the ‘true’ predisposition locus. To distinguish these possibilities, we analysed the impact of HLA‐A*02 and non‐HLA‐A*02 molecules on EBV latency‐II antigen‐specific effector CD8^+^ T cell immunity in EBV^+^cHL.

## Materials and methods

### Sample cohorts

Blood samples and diseased tissue from newly diagnosed cHL patients and blood samples from healthy participants were acquired as part of an Australasian Leukaemia and Lymphoma Group prospective observational study. EBV association was confirmed via EBV‐encoded‐RNA *in‐situ* hybridization (EBER‐ISH), as described previously 22. Peripheral blood mononuclear cells (PBMCs) were isolated and cryopreserved in 90% fetal bovine serum (FBS) with 10% dimethylsulphoxide (DMSO). This study conformed to the Declaration of Helsinki and was approved by the Human Research Ethics Committees at all participating institutions. Written informed consent was obtained in all cases.

### Digital multiplex gene expression by NanoString nCounter

Nucleic acid was extracted from 33 cHL formalin‐fixed paraffin‐embedded (FFPE) diseased node tissues (17 EBV^‐ve^cHL, 16 EBV^+^cHL) using a RecoverAll Total Nucleic Acid Extraction Kit (Life Technologies, Paisley, UK). Gene expression profiling was conducted using the nCounter platform (NanoString Technologies). All analyses were performed using NCounter software. For normalization, gene expression data were controlled internally to the mean of the positive control probes to account for interassay variability. Gene normalization was performed using the geometric mean of four housekeeper genes [phosphoglycerate kinase 1 (PGK1), glyceraldehyde‐3‐phosphate dehydrogenase (GAPDH), phosphoglycerate mutase 1 (PGAM1), ornithine decarboxylase anti‐zyme 1(OAZ1)], selected as per the manufacturer's recommendation.

### *Ex‐vivo* EBV‐specific CD8^+^ T cell responses

Peptide pools (17‐mers overlapping by 10 amino acids) were synthesized to span the entire lengths of LMP1, LMP2A and *Bam*HI Z fragment leftward open reading frame 1 (BZLF1) (Synbiosci, San Francisco, CA, USA and Mimotopes, Notting Hill, VIC, Australia). Each individual peptide in each pool was used at a final concentration of 2 μg/ml to stimulate EBV‐specific CD8^+^ T cells. Initially, PBMCs from 19 pretreatment EBV‐seropositive cHL patients (eight EBV^+^cHL, 11 EBV^–^cHL) were assayed. Subsequently, PBMCs from 14 EBV‐seropositive healthy volunteers were analysed at a separate site. PBMCs were resuspended in culture medium containing the co‐stimulatory antibodies αCD28 and αCD49d (1 μg/ml each; BD Pharmingen, Franklin Lakes, NJ, USA), brefeldin A (Golgi Plug, 10 μg/ml; BD Biosciences), monensin (Golgi Stop, 0.7 μg/ml; BD Biosciences) and fluorochrome‐labelled αCD107a. Each sample was divided into five stimulations: LMP1, LMP2A, BZLF1, an unstimulated control (co‐stimulation only) and a positive control comprising either *Staphyloccoccus* enterotoxin B (0·1 μg/ml; Sigma‐Aldrich, St Louis, MO, USA) or phorbol myristate acetate (10 ng/μl) with ionomycin (2 μg/ml). Cells were cultured overnight at 1–2 × 10^6^ cells/ml in a 37°C, 5% CO_2_ incubator. The following day, PBMCs were washed, labelled with a viability dye to enable dead cell exclusion and surface‐stained for CD3, CD4 and CD8. Cells were then fixed/permeabilized and stained intracellularly for interferon (IFN)‐γ and tumour necrosis factor (TNF)‐α. All staining procedures were conducted as described previously 23. Samples were acquired on an LSRII flow cytometer (BD Biosciences) and data analysis was performed using FlowJo version 9·2 (TreeStar Inc., Ashland, OR, USA). The gating strategy followed previously published standard practice and is shown in Supporting information, Fig. S1 24.

### LMP2A‐specific CD8^+^ T cell expansion using monogenic HLA class I cell lines

Nine healthy EBV‐seropositive HLA‐A*02 heterozygotes (three HLA‐A*01/A*02, three HLA‐A*02/A*03 and three HLA‐A*01/A*02/B*08) were tested. PBMCs were stimulated with HLA class I‐deficient, EBV‐infected 721·221 lymphoblastoid cell lines transfected separately with HLA‐A*01, A*02, A*03 or B*08. Procedures were adapted from a previously published protocol 25. Each transfected 721·221 cell line was pulsed with overlapping LMP2A peptide pools spanning the entire protein (Synbiosci). Peptides were divided into six pools (Supporting information, Table S2), each comprising 10–11 peptides at a final individual concentration of 10 μg/ml. These smaller pools at higher concentrations were used to maximize the antigen‐specific stimulus (a single pool of all 65 peptides at this high concentration would have been toxic). The corresponding unpulsed cells served as baseline controls in all experiments. Cultures were expanded for 10 days under standard conditions. The relative strength of the HLA class I‐restricted EBV‐specific CD8^+^ T cell response was assessed using intracellular cytokine staining for IFN‐γ after a 5‐h restimulation. Samples were acquired on a fluorescence activated cell sorter (FACS)Canto flow cytometer (BD Biosciences) and data analysis was performed using FlowJo version 9·2 (TreeStar Inc.).

### *In‐vitro* LMP2A‐specific CD8^+^ T cell cytotoxicity

EBV‐specific T cells were expanded by repeated stimulation with autologous EBV‐transformed B cells for 6–8 weeks using a published expansion protocol 26, 27 in four EBV‐seropositive healthy control donors (two HLA‐A*02^+^ and two HLA‐A*02^–^). Direct lysis of autologous EBV‐transformed B cells was confirmed in all cases, demonstrating successful expansion of cytotoxic EBV‐specific T cells. LMP2A‐specific cytotoxicity was quantified using autologous carboxyfluorescein succinimidyl ester (CFSE)‐labelled phytohaemagglutinin (PHA) blasts (target cells) incubated with 2 μg/ml of peptides from each of six LMP2A overlapping peptide pools (Supporting information, Table S2) in duplicate. The ratio of effector to target cells was 20 : 1. After 6 h incubation, the number of CFSE‐labelled targets remaining was determined by comparison to a constant number of CountBright absolute counting beads (ThermoFisher, Scoresby, Australia). LMP2A‐specific lysis was calculated for each well relative to the unpulsed control sample.

### HLA class I‐restricted EBV latency antigen‐specific CD8^+^ T cell responses

*Ex‐vivo* CD107ab^+^CD8^+^ T cell responses to HLA class I‐restricted peptides were assayed in 30 healthy EBV‐seropositive donors. PBMCs were resuspended in culture medium containing monensin (GolgiStop, 0.7 μg/ml; BD Biosciences), αCD107a‐fluorescein isothiocyanate (FITC) and αCD107b‐FITC (BD Pharmingen), and incubated for 5 h with peptide (2 μg/ml). Samples were acquired on a FACSCanto flow cytometer (BD Biosciences) and data analysis was performed using FlowJo version 9·2 (TreeStar Inc.). To minimize bias, an equivalent number of ‘predicted’ and ‘defined’ (31 and 30, respectively) peptides were included. The peptides were derived from LMP1/LMP2A or EBNA3A/3B/3C and restricted by one of the following HLA class I allotypes: HLA‐A*01, A*02, A*03, A*11, A*24, B*07, B*08, B*35, B*37, B*44 or B*60 (B*40:01). Equivalent numbers of defined epitopes covering comparable numbers of HLA class I alleles were used for the immunodominant EBNA3A/3B/3C (15 peptides, six alleles) and the subdominant LMP1/LMP2A (15 peptides, seven alleles) proteins. Combined, these alleles cover 75% of HLA‐A and 57% of HLA‐B alleles in the Australian population 28. Peptides (and references) are listed in Supporting information, Table S3. All defined epitopes were validated previously in functional assays. For HLA class I alleles with few or no known epitopes, predicted peptides were identified *in silico* (http://www.syfpeithi.de) using a previously described algorithm [29]. The ‘SYFPEITHI’ scores of defined epitopes were used to delineate a threshold score for the predicted peptides.

### HLA class I peptide binding of algorithm‐predicted peptides

Binding of predicted HLA‐A*03‐restricted peptides was confirmed using HLA‐A*03‐expressing T2 cell lines, as described previously 30. Expression of stabilized HLA‐A*03 on the cell surface after peptide pulsing was assessed by flow cytometry. Results are reported as the mean fluorescence intensity above the negative (no peptide) control.

Binding of predicted HLA‐A*01‐restricted peptides was confirmed by *in‐vitro* HLA‐A*0101‐peptide complex refolding and thermal stability analysis by circular dichroism (CD) spectroscopy. Competent Rosetta DE3 *Escherichia coli* cells were used to produce the HLA‐A*0101 and beta 2‐microglobulin (β2M) chains, as described previously 31. For a 1‐l refold, 30 mg of HLA‐A*0101 was mixed with 30 mg of β2M and 4 mg of peptide at 37°C for 15 min. The mixture was then added to cold refold buffer [50 mM Tris pH 8, 2 mM ethylenediamine tetraacetic acid (EDTA), 400 mM L‐arginine, 6 mM cysteamine hydrochloride and 4 mM cystamine]. Refolds were stirred at 4°C for > 1 h. Dialysis was carried out against 10 mM Tris pH 8.1 until the conductivity of the refolds was <2 mS/cm. The refolds were then filtered, purified by ion exchange using a Poros50HQ^TM^ column and gel filtered into phosphate‐buffered saline (PBS) using a Superdex200HR^TM^ column. Protein quality was analysed by Coomassie‐stained sodium dodecyl sulphate‐polyacrylamide gel electrophoresis (SDS‐PAGE). After ion exchange, the central five fractions around the maximum HLA class I peak (by SDS gel) from each refold were selected for gel filtration. After gel filtration, the central four fractions around the maximum HLA class I peak from each refold were selected for analysis by spectrometry (A280 nm) to determine the final protein yield. Two positive control peptides from cytomegalovirus, VTEHDTLLY 32 and YSEHPTFTSQY 25, and a negative control peptide with a binding sequence optimal for HLA‐A*0201 (ALAAAAAAV), were used in addition to the 13 predicted HLA‐A*01‐restricted EBV‐derived peptides.

Thermal stability of HLA‐A*0101/β2M/peptide complexes was assessed by CD spectroscopy. Data were collected on an Aviv Model 215 spectropolarimeter (Aviv Biomedical Inc., Lakewood, NJ, USA) equipped with a Peltier thermostatted cell holder using a 0·1‐cm quartz cell. Proteins were dissolved in 75 mM NaCl, 20 mM ${\text{PO}}_{4}^{–}$, pH 7·5. Melting curves were recorded in 0·5°C intervals from 4°C up to a maximum temperature when protein aggregation was observed with settings resulting in an average heating rate of ∼30°C/h. Values were corrected to a calibration curve recorded with the temperature measured in the cell. Melting curves were analysed assuming a two‐state trimer‐to‐monomer transition from the native (N) to unfolded (U) conformation N_3_ ↔ 3U with an equilibrium constant K = [U]^3^/[N_3_] = F/[3c^2^ (1‐F)^3^], where F and c are the degree of folding and protein concentration, respectively. Data were fitted as described 33 using the non‐linear least‐squares routine of Origin version 7·5 (OriginLab, Northampton, MA, USA). Fitted parameters were the melting temperature T_M_, at which 50% of proteins are in the folded and unfolded state, van't Hoff's enthalpy ΔH_vH_ at the transition midpoint and the slope and Θ‐intercept of the native baseline assumed as a linear function of the temperature. As all protein complexes aggregated at various degrees of unfolding, the ellipticity of the unfolded state was set as a constant of −4400 degrees cm^2^ dmol^−1^; this value resulted from fitting melting curves of LMP2A_LTE_ and positive control peptide VTEHDTLLY‐containing complexes, which showed the least aggregation tendency, and is in good agreement with values reported for other thermally denatured proteins 34. For all peptides, the coefficient of determination for fitted curves *versus* measurements was *r* ^2^ > 0·99.

### Statistical analysis

Combined (but not summed) IFN‐γ, TNF‐α and CD107a responses to EBNA1/LMP1/LMP2A/BZLF1 in patients with cHL and healthy volunteers were compared using a linear mixed‐effects model (with a random effect for subject). This enabled comparison of groups while accounting for the correlation induced by measuring multiple (but not necessarily comparable) responses in the same individual 35. Using spice (software version 5·3, downloaded from http://exon.niaid.nih.gov), individual and polyfunctional IFN‐γ, TNF‐α and CD107a responses in patients with cHL were compared by Wilcoxon's signed‐rank tests and a partial permutation test, as described previously 36. Healthy donor *ex‐vivo* CD107ab^+^CD8^+^ T cell responses against defined and predicted HLA class I‐restricted peptides from LMP1/LMP2A and EBNA3A/3B/3C were compared using the Mann–Whitney *U*‐test. In cases where responses against multiple peptides were measured in the same individual, individual responses were considered independent. Significance above zero was determined using a one‐sample *t*‐test with one‐tailed *P*‐values. Cytotoxicity was compared using Fisher's exact test with a two‐tailed *P*‐value. Statistical analysis was performed using GraphPad Prism version 5·0 (Graphpad Software Inc., San Diego, CA, USA) and spss statistics version 19 (IBM, Armonk, NY, USA).

## Results

### Intratumoral CD8 : LMP2A ratios are enriched in HLA‐A*02^–^ EBV^+^cHL

**Figure 1:**
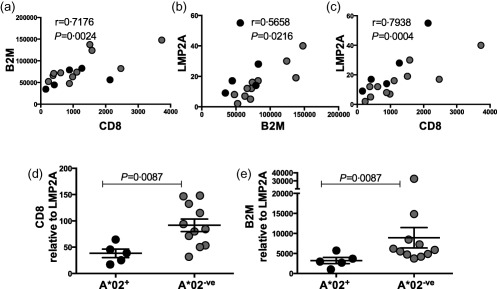
Gene expression in Epstein–Barr virus (EBV)^+^ classical Hodgkin lymphoma (cHL) diseased tissue. (a) Correlation of beta 2‐microglobulin (β2M) and CD8 expression levels. (b) Correlation of latent membrane protein (LMP)2A and β2M expression levels. (c) Correlation of LMP2A and CD8 expression levels. Spearman's correlation = *r*. (d) CD8 expression relative to LMP2A in human leucocyte antigen (HLA)‐A*02^+^ compared to HLA‐A*02^–^ patients. (e) β2M expression relative to LMP2A in HLA‐A*02^+^ compared to HLA‐A*02^–^ patients. Error bar represents standard error of the mean. Solid black circles represent HLA‐A*02^+^ patients. Grey circles represent HLA‐A*02^–^ patients.

Gene expression was quantified in 33 cHL diseased node FFPE tissues (17 EBV^–^cHL, 16 EBV^+^cHL) using NanoString nCounter 37. The expression levels of CD8 and β2M as markers of immune effector and antigen presentation, respectively, and the EBV latency II genes (EBNA1, LMP1 and LMP2A with the EBV‐lytic gene BZLF1 as a comparator) were measured. CD8 expression correlated with β2M in the entire cHL cohort (*r* = 0·5906, *P* = 0·0003), consistent with intratumoral CD8 being proportional to antigen presentation. In the EBV^+^cHL subgroup, the β2M–CD8 correlation was more marked (*r* = 0·7176, *P* = 0·0024, Fig. 1a). In this subgroup, β2M also correlated with LMP2A levels (*r* = 0·5685, *P* = 0·0216, Fig. 1b). Accordingly, CD8 expression correlated with LMP2A (*r* = 0·7938, *P* = 0·0004, Fig. 1c). No correlations were found for the other EBV genes with either CD8 or β2M, and there was no difference in expression of CD8 or β2M between EBV^+^ and EBV^–^cHL.

Both EBNA1 and LMP1 are known to encode for antigenic peptides that expand IL‐10‐secreting regulatory CD4^+^ T cells 38, 39. However, no correlations were found between the EBV latency II genes and expression of either CD4 or the regulatory T cell markers IL‐10, lymphocyte‐activation gene 3 (LAG3) and forkead box protein 3 (FoxP3) [*P* = not significant (n.s.)].

Comparisons of HLA‐A*02^+^ (*n =* 5) and HLA‐A*02^–^ (*n =* 11) patients with EBV^+^cHL showed no difference in expression levels of β2M, CD8, LMP2A or any of the other EBV genes. However, CD8 and β2M (normalized to the expression of LMP2A) were elevated markedly in HLA‐A*02^–^ *versus* HLA‐A*02^+^ patients (*P* = 0·0087, *P* = 0·0087 Fig. 1d,e). This was not observed when expression was normalized to the other EBV latency II genes. These results demonstrate that, for a given LMP2A expression level, there is more CD8 and β2M expression within the malignant lymph node in HLA‐A*02^–^ compared to HLA‐A*02^+^ patients with EBV^+^cHL.

### HLA‐A*02^+^ patients with EBV^+^cHL exhibit increased responses to EBV latency‐II antigens relative to HLA‐A*02^–^ patients

The observation that CD8 is relatively enriched within HLA‐A*02^–^ EBV^+^cHL nodes suggests that EBV latency‐II‐specific effector CD8^+^ T cell immunity is relatively ineffective in these patients. To investigate the impact of HLA‐A*02 on EBV latency‐II‐specific effector CD8^+^ T cell immunity, a series of functional assays were performed. Initially, we tested CD8^+^ T cell immunity against relevant EBV latency antigens in pretherapy cHL patients, with EBV tissue status and HLA class I as covariates. Previous studies in cHL have not evaluated the contribution of non‐defined subdominant epitopes to the total CD8^+^ T cell response or assessed whether HLA class I alleles impact the magnitude of the response 40, 41. Effector molecule testing in these studies was also limited to IFN‐γ production.

**Figure 2:**
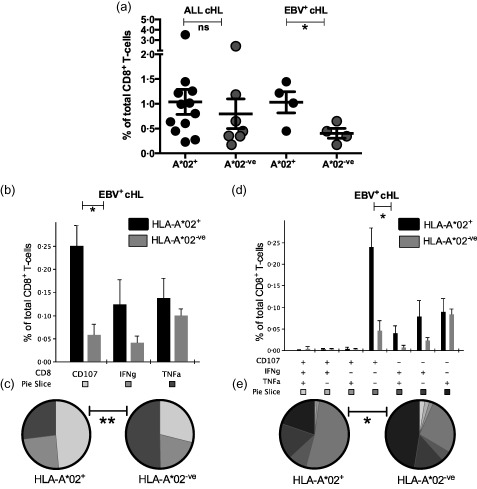
*Ex‐vivo* Epstein–Barr virus (EBV)‐specific CD8^+^ T‐cell responses in patients with classical Hodgkin lymphoma (cHL) . (a) Summed percentages of *ex‐vivo* latent membrane protein (LMP)1/2A‐specific CD8^+^ T cell responses defined by interferon (IFN)‐γ, tumour necrosis factor (TNF)‐α and CD107a. Error bars represent standard error of the mean. *P*‐values were calculated using a linear mixed effects model. (b–c) Comparison of individual effector molecules in human leucocyte antigen (HLA)‐A*02^+^ versus HLA‐A*02^‐ve^ patients with EBV^+^cHL. (b) Percentage of total CD8^+^ T cells specific for LMP1/2A. (c) Percentage of the total LMP1/2A‐specific CD8^+^ T cell subset. (d–e) Comparison of polyfunctional LMP1/2A‐specific CD8^+^ T cell responses in HLA‐A*02^+^ *versus* HLA‐A*02^‐ve^ patients with EBV^+^cHL. (d) Percentage of total CD8^+^ T cells specific for LMP1/2A. (e) Percentage of the total LMP1/2A‐specific CD8^+^ T cell subset. **P < 0·01; *P < 0·05; *P* > 0·05 = not significant.

First, we tested 19 cHL patients (Table 1). Blood samples were collected at diagnosis prior to therapy. Age and gender were not significantly different between subgroups, which were also comparable for Ann Arbor stage and prognostic score 42. To ensure that multi‐faceted CD8^+^ effector T cell immunity was assayed, we used polychromatic flow cytometry to measure *ex‐vivo* IFN‐γ and TNF‐α production together with CD107a mobilization. Overlapping peptide pools spanning EBNA1 and the latent proteins LMP1 and LMP2A were used to ensure comprehensive antigenic coverage. A peptide pool spanning the EBV lytic protein BZLF1 (not expressed by EBV^+^ HRS cells) was included as a comparator. Peptide stimulations were conducted overnight to enhance sensitivity, and highly stringent gating strategies were employed to maximize specificity (Supporting information, Fig. S1). A linear mixed‐effects model was used to incorporate and compare all three effector markers and any compounding significance while accounting for multiple measurements within a given individual (represented in Fig. 2a as summed percentages of IFN‐γ, TNF‐α and CD107a responses). spice software, designed specifically for post‐cytometric complex multivariate data sets, was used to conduct Wilcoxon signed‐rank tests and permutation comparisons on individual (Fig. 2b,c) and polyfunctional (Fig. 2d,e) responses.

**Table 1:** Characteristics of classical Hodgkin lymphoma (cHL) patients tested *ex vivo* for CD8^+^ T cell responses

Patient no.	EBER‐ISH	Age	Gender	‘B’ symptoms	Stage	Prognostic score*	Classical subtype	HLA‐A*02	HLA class I
1	Negative	79	F	Yes	III	3	NS	Negative	A*01/*30, B*51/*62
2	Negative	25	M	Yes	IIIb	3	NS	Negative	A*11/*11, B*60/*60
3	Negative	79	F	No	III	1	NS	Negative	A*01/*31, B*57/*61
4	Negative	33	F	Yes	IIb	2	LD	Negative	A*02/*25, B*62/*63
5	Negative	55	M	No	II	3	MC	Positive	A*02/*03, B*13/*51
6	Negative	54	M	Yes	IVb	5	NS	Positive	A*02/*31, B*44/*60
7	Negative	22	M	Yes	IVb	5	Classical unspecified	Positive	A*01/*02, B*35/*57
8	Negative	21	M	No	IV	3	NS	Positive	A*02/*03, B*07/*51
9	Negative	27	M	No	III	3	NS	Positive	A*02/*03, B*07/*51
10	Negative	20	F	No	IVa	1	Classical unspecified	Positive	A*02/*02, B*07/*35
11	Negative	20	F	Yes	IIIb	3	NS	Positive	A*01/*02, B*08/*49
12	Negative	18	M	No	III	2	NS	Negative	A*11/*24, B*18/*60
13	Positive	48	M	No	II	2	LR	Negative	A*24/*24, B*18/*51
14	Positive	40	M	No	II	1	MC	Negative	A*01/*24, B*08/*51
15	Positive	40	M	Yes	IVb	5	NS	Negative	A*01/*03, B*35/*75
16	Positive	33	M	Yes	II	2	NS	Positive	A*02/*29, B*07/B*44
17	Positive	25	F	No	IIa	0	NS	Positive	A*02/*02, B*44/*44
18	Positive	49	M	No	IV	4	NS	Positive	A*01/*02, B*37/*57
19	Positive	21	F	No	IIa	1	NS	Positive	A*02/*66, B*41/*44

a *Hasenclever 42. LD = lymphocyte‐depleted; MC = mixed‐cellularity; NS = nodular sclerosing; LR = lymphocyte‐rich; M = male; F = female; HLA = human leucocyte antigen; EBER‐ISH = Epstein–Barr virus‐encoded‐RNA *in‐situ* hybridization.

Global effector CD8^+^ T cell responses (defined as combined IFN‐γ, TNF‐α and CD107a) were compared for the EBV latency II proteins (EBNA1, LMP1, LMP2A). Responses were analysed as total EBV latency II (summed EBNA1, LMP1/2A‐specific), LMP1/2A (summed LMP1/2A‐specific) and individual protein responses. No significant differences were found between patients with EBV^+^cHL and EBV^–^cHL. Furthermore, response magnitudes across all patients with cHL independent of EBV tissue status were equivalent irrespective of HLA‐A*02 expression (Fig. 2a). In addition, the relevant EBV‐specific CD8^+^ T cell responses in patients with EBV^–^cHL were not influenced by HLA‐A*02 status.

Interestingly, the combined IFN‐γ, TNF‐α and CD107a CD8^+^ T cell responses were significantly greater in HLA‐A*02^+^ compared to HLA‐A*02^‐ve^ patients with EBV^+^cHL for the LMP1/2A proteins (*P* = 0·0371, Fig. 2a). Although elevated response levels were seen in HLA‐A*02^+^ EBV^+^cHL patients for total EBV latency II (*P* = 0·0753) and individual EBNA1, LMP1 and LMP2A proteins (*P* = 0·3329, *P* = 0·0525 and *P* = 0·0983, respectively), these did not reach significance.

We then compared individual immune effector molecules stratified by HLA‐A*02 status using spice. Although all three functional markers were elevated in HLA‐A*02^+^ compared to HLA‐A*02^–^ patients with EBV^+^cHL for LMP1/2A proteins (CD107a, 4·3‐fold; IFN‐γ, 3·0‐fold; TNF‐α, 1·4‐fold), only CD107a reached significance (*P* = 0·007, Fig. 2b). Across all LMP1/2A‐specific effector CD8^+^ T cells, the proportion of individual effector molecules was significantly different between HLA‐A*02^+^ and HLA‐A*02^–^ patients (*P* = 0·0041, Fig. 2c). By polyfunctional analysis, this significant increase was attributable to CD107a single‐positive CD8^+^ T cells (CD107a^+^IFN‐γ^‐^TNF‐α^‐^; *P* = 0·007, Fig. 2d) and the proportion of polyfunctional immune effectors within all LMP1/2A‐specific CD8^+^ T cells was also significantly different (*P* = 0·043, Fig. 2e). In HLA‐A*02^+^ EBV^+^cHL patients, the majority of immune effector cells were CD107a single‐positive CD8^+^ T cells. No differences (*P* = n.s.) were observed for BZLF1‐specific CD8^+^ T cell responses between any patient categories.

In line with our findings that differences between HLA‐A*02^+^ and HLA‐A*02^–^ patients were detectable only in EBV^+^cHL, analysis of 14 healthy age‐/sex‐matched controls (using the linear mixed‐effects model to evaluate comprehensive CD8^+^ T cell responses to the EBV latency‐II proteins) revealed no significant differences between HLA‐A*02^+^ (*n =* 7) and HLA‐A*02^–^ (*n =* 7) individuals (Supporting information, Fig. S2a). In patients with cHL, global effector CD8^+^ T cell responses were greater for LMP2A compared with LMP1 (*P* = 0·016) and EBNA1 (*P* < 0·0001) (Supporting information, Fig. S2b). Similarly, for healthy controls global effector CD8^+^ T cell responses were greater for LMP2A compared with LMP1 (*P* = 0·011) and EBNA1, although the latter did not reach statistical significance (Supporting information, Fig. S2c).

Thus, by *ex‐vivo* analysis, we observed modest differences in CD8^+^ T cell responses against relevant EBV latency‐II proteins between HLA‐A*02^+^ and to HLA‐A*02^–^ patients with EBV^+^cHL. These results were not observed in EBV^–^cHL patients or healthy participants. In line with our tissue expression data, these results suggest that EBV latency‐II‐specific effector CD8^+^ T cell immunity may be less effective in HLA‐A*02^–^ EBV^+^cHL.

### LMP2A‐specific responses restricted by HLA‐A*02 achieve greater magnitudes than those restricted by other HLA class I molecules

*Ex‐vivo* assays are less sensitive than *in‐vitro* expansion for the detection of low‐frequency EBV‐specific CD8^+^ T cells 43. Indeed, low‐frequency responses can be detected via *in‐vitro* expansion in patients receiving immunosuppressive therapies 13. Furthermore, up to four different HLA‐A/B molecules can potentially present relevant EBV‐derived epitopes in each individual, adding a confounding layer of complexity to single allele‐based effects.

To overcome these limitations, we generated mutant HLA class I‐negative 721·221 cell lines transfected individually with HLA‐A*01, HLA‐A*02, HLA‐A*03 and HLA‐B*08. Previous work with these cell lines demonstrated an absence of HLA‐A*01‐restricted responses to endogenously processed EBV latency antigens. However, this system was biased preferentially towards the immunodominant EBNA3A/3B/3C antigens that are not expressed by HRS cells 25. In order to use this single allele system to evaluate responses relevant to the EBV^+^cHL setting, transfected 721·221 cells were pulsed with LMP2A peptides. LMP2A was chosen for this purpose, as it is the most immunogenic of the three EBV genes expressed in EBV^+^cHL and because it was the only gene we found to associate significantly with HLA‐A*02 in diagnostic tissues. Overlapping peptides divided into six pools spanning the entire LMP2A protein were used to ensure inclusion of the total CD8^+^ T cell response restricted by each HLA class I molecule, regardless of the specific target epitopes (Supporting information, Table S2). PBMCs from healthy EBV‐seropositive participants were stimulated separately with each peptide pool to expand LMP2A‐specific CD8^+^ T cells. The results were summed for each allele. Nine EBV‐seropositive donors were tested, all heterozygous for HLA‐A*02. Other HLA class I donor alleles were selected based on the literature. Notably, HLA‐A*01 has been associated consistently with an increased incidence of EBV^+^cHL 15, 16, 17. HLA‐B*08 is known to be in linkage disequilibrium with HLA‐A*01 44, and a functionally defined HLA‐B*08‐restricted LMP2A‐derived epitope was identified recently 45. Finally, HLA‐A*03 was chosen as a relatively common HLA class I allele in Australia. Together, these four alleles represent > 75% of the Australian population 46. Accordingly, donors 1–3 were HLA‐A*01/A*02, donors 4–6 were HLA‐A*02/A*03 and donors 7–9 were HLA‐A*01/A*02/B*08.

**Figure 3:**
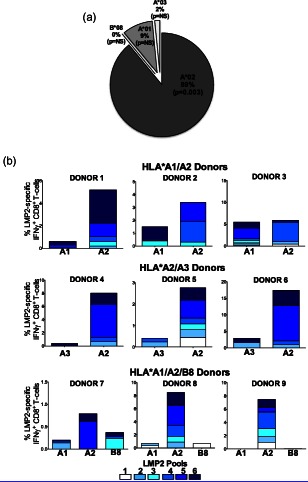
Human leucocyte antigen (HLA) class I‐restricted expansion of latent membrane protein (LMP)2A‐specific interferon (IFN) γ^+^CD8^+^ T cells. (a) Percentages of total HLA class I‐restricted LMP2A‐specific CD8^+^ T cell responses generated *in vitro* from healthy Epstein–Barr virus (EBV) seropositive HLA‐A*02 heterozygotes (*n =* 9). Only HLA‐A*02‐restricted responses were significantly above zero. (b) The combined expansion of LMP2A‐**s**pecific IFN‐γ^+^CD8^+^ T‐cells is shown for all donors from the three HLA‐A*02 heterozygote combinations. Each bar depicts the percentage of LMP2A‐specific IFN‐γ^+^CD8^+^ T cells expanded from one of the six peptide pools. The peptide pools are described in Supporting information, Table S2.

IFN‐γ‐specific CD8^+^ T cells inhibit *in‐vitro* transformation of EBV‐infected B cells 47, 48. The cytokine may also play a critical role in the pathogenesis of cHL via the canonical Janus kinase–signal transducer and activator of transcription (JAK–STAT) pathway known to be over‐expressed in HRS cells 49. Therefore, identifying any differences in IFN‐γ‐specific CD8^+^ T cell responses has important implications. *In‐vitro* expansion permitted more sensitive discrimination of the impact of HLA class I status on IFN‐γ^+^ responses. As shown in Fig. 3, HLA‐A*02 uniformly expanded more LMP2A‐specific IFN‐γ^+^ CD8^+^ T cells. The total response for each HLA class I allele (i.e. all HLA‐A*01, all HLA‐A*02, all HLA‐A*03 and all HLA‐B*08) was significantly greater than zero only for HLA‐A*02 (*P* = 0·0034). Thus, in an *in‐vitro* system with few confounding influences, HLA‐A*02 generated more LMP2A‐specific IFN‐γ^+^ CD8^+^ T cells compared to HLA‐A*01, HLA‐A*03 and HLA‐B*08.

Of note, peptide pool 5 generated the greatest response (37% of the total HLA‐A*02‐restricted responses) and contained the sequences of two known HLA‐A*02 epitopes (FLYALALLL, LLWTLVVL) 12. Peptide pool 6 contained the sequences of three known HLA‐A*02 epitopes (CLGGLLTMV, LTAGFLIFL and LLSAWILTA) and generated 22% of the total HLA‐A*02‐restricted responses. Peptide pool 4 also generated 22% and contained the sequence of an identified HLA‐A*02 epitope (GLGTLGAAL). Pools 1–3 did not contain known HLA‐A*02 epitopes and generated 4, 8 and 7% of the total HLA‐A*02‐restricted responses, respectively.

To confirm that functional LMP2A‐specific responses restricted by HLA‐A*02 achieve greater magnitudes than those restricted by other HLA class I molecules in conventional cytotoxicity assays, we took advantage of a published expansion protocol that generates EBV‐specific CD8^+^ T cells for therapeutic use 26, 27. This method uses autologous EBV‐transformed B cells as antigen‐presenting cells, thus not only is it physiologically relevant but it expands a broad range of EBV latent antigen‐specific CD8^+^ T cells across multiple HLA class I alleles. The latter is an important point, because it permits a comparison of HLA‐A*02 *versus* non‐HLA‐A*02 LMP2A‐specific CD8^+^ T cells in a setting in which other EBV latent antigens, including those presented by other HLA class I alleles, are also presented. Consistent with earlier results, minimal killing was observed in non‐HLA‐A*02 participants (<5% mean LMP2A‐specific killing), whereas strong killing was observed in HLA‐A*02 individuals (>54% mean LMP2A‐specific killing; *P* < 0·0001; Supporting information, Fig. S3).

### Prediction and verification of HLA class I‐binding EBV latency antigen‐derived peptides

Next, we assessed the impact of multiple HLA class I alleles on the hierarchy of CD8^+^ T cell responses against the spectrum of EBV latency proteins. For immunodominant proteins, we tested EBNA3A/3B/3C (very few EBNA2 and EBNA‐LP epitopes have been identified) 50. Based on our earlier findings, subdominant proteins were restricted to LMP1/LMP2A and not EBNA1. Initially, we selected ‘defined’ epitopes shown previously to elicit CD8^+^ effector T cell immunity. For EBNA3A/3B/3C proteins, several such epitopes presented by a variety of HLA class I molecules are known. By contrast, for LMP1/LMP2A, there are relatively few defined HLA class I epitopes, with the exception of peptides presented by HLA‐A*02 12, 51, 52. As HLA‐A*01, HLA‐A*03 and HLA‐B*37 have each been associated with differential susceptibility to EBV^+^ lymphomas 15, 16, 17, 53, we aimed to increase the number of defined EBV latency antigen‐derived epitopes restricted by these HLA class I molecules. To achieve this, we screened for potential HLA class I binding peptides using the bioinformatic algorithm ‘SYFPEITHI’ 29. Thirty functionally defined epitopes (Supporting information, Table S3) were scored by the algorithm in order to define the parameters by which predicted peptides were to be selected. Defined epitopes had a ‘SYFPEITHI’ score ranging from 7 to 36 (mean: 23·4). To reduce the likelihood of predicted peptides being unable to bind to the relevant HLA class I molecule, we selected a cut‐off value that excluded the bottom 20th centile of the defined epitopes (‘SYFPEITHI’ score: ≥ 21). A total of 31 predicted peptides were used with HLA class I peptide binding scores ≥ 21 (range = 21–31; mean score = 26·1).

**Figure 4:**
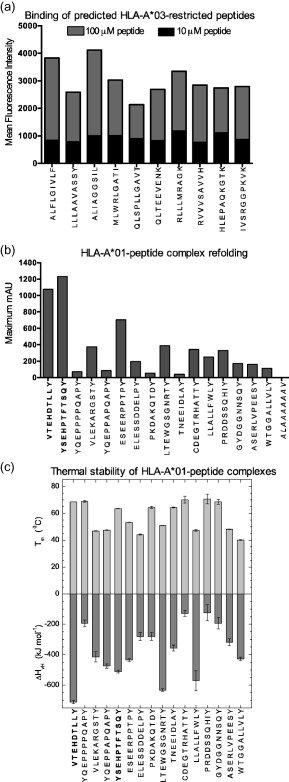
Human leucocyte antigen (HLA) class I peptide binding assays for algorithm‐predicted peptides. (a) HLA‐A*03 binding of predicted peptides was confirmed using a T2 cell line transfected with HLA‐A*03. Peptides were added at concentrations of 10 μM or 100 μM, and HLA class I expression was measured by flow cytometry analysis using an αHLA‐A*03 antibody. Data are shown as the mean fluorescence intensity above the negative control. (b) HLA‐A*01 binding of predicted peptides was confirmed by *in‐vitro* refolding. Maximum mAU values are shown. Positive control peptides are in shown in bold type. The negative control peptide is shown in italics. (c) Thermal stability with respect to melting temperature (upper panel) and van't Hoff's enthalpy of unfolding (lower panel). Error bars represent standard error of the respective parameter based on fitting each set of measured data. Positive control peptides are shown in bold type.

**Figure 5:**
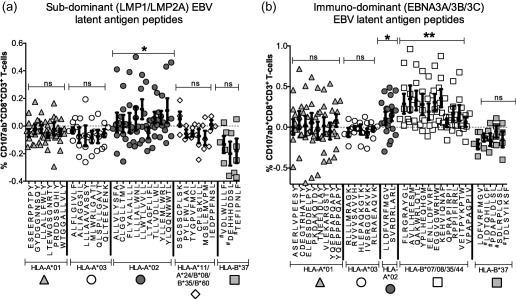
Hierarchy of *ex‐vivo* human leucocyte antigen (HLA) class I‐restricted CD8^+^ T cell responses to known and predicted peptides derived from latent membrane protein (LMP)1/LMP2A and EBNA3A/3B/3C. The *ex‐vivo* CD107ab^+^CD8^+^ T cell responses to individual peptides are shown. Complete epitope sequences are listed together with the restricting allotypes; further details are shown in Supporting information, Table S3. Error bars represent standard error of the mean. #Predicted HLA‐B*37‐restricted peptides with unconfirmed binding. (a) Subdominant LMP1/LMP2A‐derived epitope‐specific responses. (b) Immunodominant EBNA3A/3B/3C‐derived epitope‐specific responses. The *P*‐values indicate significance of combined peptide responses above zero: **P < 0·01; *P < 0·05; *P* > 0·05 = not significant.

Binding of predicted HLA‐A*03‐restricted peptides was confirmed using the HLA class I stabilization assay on live T2 cells, which lack the transporter associated with antigen processing, transfected with HLA‐A*03 (Fig. 4a) 30. Using this approach, we confirmed that all 10 predicted peptides bound HLA‐A*03. As no stable HLA‐A*01‐transfected T2 cell line was available, binding was confirmed for all predicted HLA‐A*01‐restricted peptides using *in‐vitro* HLA‐A*0101‐peptide refolding and thermal stability CD analysis. The maximum mAU at 280 nm for each 1l HLA‐A*0101 refold is shown in Fig. 4b. For all peptides, the purity of the peak containing properly refolded HLA‐A*0101‐peptide was confirmed by SDS gel electrophoresis. All the peptides, with the exception of the negative control, generated detectable amounts of conformationally intact HLA‐A*0101. Melting temperatures (T_M_) ranged between 40°C and 72°C (Fig. 4c), indicating that all the HLA‐A*0101‐peptide variants were stable at or above body temperature. Values for van't Hoff's enthalpy of unfolding ranged from −110 to −700 KJ mol^−1^, with more negative values indicating a higher ratio of folded to misfolded peptide. Consistent with the indications from the refolding data, the positive control peptides and LMP2A_ESE_ were the most stable complexes overall (i.e. highest overall T_M_ range and most negative van't Hoff's enthalpy of unfolding values). Peptides LMP2A_LTE_ and LMP1_LLA_ were also towards the high end of the stability range. Although the other peptides tested demonstrated a range of different stabilities, all the HLA‐A*0101‐peptide variants generated a CD signal consistent with the presence of properly refolded HLA class I. The predicted HLA‐B*37‐restricted peptides are identified in Supporting information, Table S3 and Fig. 5, but HLA class I binding was not tested directly, as neither the *in‐vitro* HLA‐B*37‐peptide refolding system nor a stable HLA‐B*37‐transfected T2 cell line was available.

### LMP1/LMP2A‐specific CD8^+^ T cell responses are confined to HLA‐A*02

The defined and predicted peptide epitopes listed in Supporting information, Table S3 were used to investigate the HLA class I hierarchy of *ex‐vivo* effector CD8^+^ T cell responses to immunodominant and subdominant EBV latency proteins. Blood samples were obtained for this purpose from 30 healthy participants (mean age = 44 years; female/male ratio: 15 : 15). CD107 was selected as the single functional readout, as it was the only functional marker to reach significance in the previous *ex‐vivo* assays reported above. As we were testing for individual and not pooled (total) peptide responses, CD107b was combined with CD107a to enhance sensitivity.

Initially, we compared CD107ab^+^CD8^+^ T cell responses against 30 subdominant LMP1/LMP2A peptides (15 predicted and 15 defined, presented by nine different HLA class I allotypes; Fig. 5a). The defined epitopes were presented by HLA‐A*02, A*11, A*24, B*08, B*35 or B*60. Each of these alleles is present at a carrier frequency of > 10% in the Australian population. The predicted peptides were presented by HLA‐A*01, A*03 or B*37. The rationale for focusing on these three HLA class I alleles has already been outlined. Within the Australian population, the total frequencies of HLA‐A and HLA‐B allotypes that restrict these peptides (defined and predicted) are ∼75 and ∼33%, respectively.

We were unable to detect *ex‐vivo* responses significantly above zero for any of the individual peptides, regardless of HLA class I restriction (Fig. 5a). However, by grouping the individual peptide responses into the total response for each HLA class I allele, we found that the HLA‐A*02‐restricted responses were significantly greater than zero (*P* = 0·016). This was not observed for HLA‐A*01, HLA‐A*03, HLA‐B*37 or the group of defined peptides for HLA‐A*11, A*24, B*08, B*35 and B*60. These results indicate that, despite their modesty in the *ex‐vivo* setting, HLA‐A*02‐restricted responses are dominant for LMP1/LMP2A.

Next, we compared *ex‐vivo* CD8^+^ T cell responses to the immunodominant EBV latency‐III proteins EBNA3A/3B/3C, which are not expressed in EBV^+^cHL. To keep the analysis comparable with EBV latency‐II antigens, we used 31 peptides (16 predicted and 15 defined, presented by eight different allotypes; Fig. 5b). As with LMP1/LMP2A, we included peptides presented by HLA‐A*01, A*02, A*03 and B*37, including a defined EBNA3A‐derived HLA‐A*03‐restricted epitope and a defined EBNA3C‐derived HLA‐B*37‐restricted epitope. Other defined EBNA3A/3B/3C‐derived epitopes were presented by HLA‐B*07, B*08, B*35 or B*44, which account for ∼50% of HLA‐B alleles in Australian populations.

As shown in Fig. 5b, we were able to detect responses to EBNA3A/3B/3C that were significantly greater than zero for one of two HLA‐A*02‐restricted peptides (EBNA3A_SVR_) and five of 11 HLA‐B*07/B*08/B*35/B*44‐restricted peptides (EBNA3A_FLR, QAK, YPL_, EBNA3C_EGG, KEH_). None of the other alleles generated responses significantly greater than zero. The total responses to peptides presented by HLA‐A*02 and HLA‐B*07/B*08/B*35/B*44 were both significantly greater than zero (*P* = 0·0126 and *P* < 0·0001, respectively). The magnitudes of the HLA‐B*07/B*08/B*35/B*44‐restricted EBNA3A/3B/3C‐specific responses were significantly greater than the HLA‐A*02‐restricted EBV latency‐II responses (*P* = 0·0008).

In summary, we were able to detect *ex‐vivo* responses to EBNA3A/3B/3C‐derived peptides restricted by several HLA class I allotypes. By contrast, responses to EBV latency‐II peptides were detected only in the context of HLA‐A*02. Thus, the immunodominant EBV latency‐III proteins generate strong CD8^+^ T cell responses through a broad range of HLA class I allotypes, whereas the subdominant proteins expressed in EBV^+^cHL generate modest responses through a single common allotype, namely HLA‐A*02.

## Discussion

In this study, we investigated the immunopathology underpinning the decreased frequency of HLA‐A*02 carriers observed in EBV^+^cHL. The results indicate that this is not a surrogate association, but rather reflects the impact of HLA class I on the established hierarchy of CD8^+^ T cell responses against EBV latency‐II antigens. To reach these conclusions, we used a series of distinct but complementary approaches.

Our initial investigation assessed relevant gene expression in the diseased tissues of cHL patients. Intriguingly, we observed a strong correlation between LMP2A and CD8 in diagnostic EBV^+^cHL lymph nodes. Importantly, for a given level of LMP2A, intratumoral CD8 expression was higher in HLA‐A*02^‐ve^ compared to HLA‐A*02^+^ EBV^+^cHL patients. These data imply that CD8^+^ T cell anti‐tumoral immunity is relatively ineffective in HLA‐A*02^–^ EBV^+^cHL. This is consistent with a study that found intratumoral LMP2A‐specific CD8^+^ T cells difficult to detect in EBV^+^cHL 40.

To test if the presence of HLA‐A*02 impacts anti‐tumoral immunity in EBV^+^cHL, we assessed *ex‐vivo* responses to relevant EBV latency antigens in pretherapy cHL patients. In order to obtain an unbiased and more comprehensive assessment of CD8^+^ T cell immunity with high sensitivity and specificity, we used overlapping EBNA1/LMP1/LMP2A peptide pools and measured both cytokine production (IFN‐γ and TNF‐α) and degranulation (CD107a) 35, 36. Even a modest cohort size was sufficient to observe significantly reduced LMP1/LMP2A antigen‐specific CD8^+^ T cell immunity in newly diagnosed HLA‐A*02^–^ *versus* HLA‐A*02^+^ EBV^+^cHL patients. No differences between HLA‐A*02^+^ and HLA‐A*02^–^ immune responses were observed in either EBV^–^cHL patients or healthy participants. These findings suggest that diminished immune‐surveillance as a result of HLA class I hierarchies may be pertinent only to the pathogenesis of EBV^+^cHL. Although this approach enabled an unbiased evaluation of relevant anti‐tumoral immunity in EBV^+^cHL, *ex‐vivo* assays are less sensitive than *in‐vitro* expansion for the detection of low‐frequency EBV‐specific CD8^+^ T cells 43. Furthermore, these results were confounded by the presence of additional HLA‐A/B molecules potentially masking single allele‐based effects.

To overcome these limitations and specifically test the impact of individual HLA class I alleles on relevant EBV‐specific CD8^+^ T cell immunodominance hierarchies, peptide‐pulsed single HLA class I‐restricted cell lines were used to expand LMP2A‐specific CD8^+^ T cells *in vitro* from EBV‐seropositive healthy HLA‐A*02 heterozygotes. *In‐vitro* expansion would permit more sensitive discrimination of the impact of HLA class I status on CD8^+^ T cell immunodominance hierarchies than *ex‐vivo* analysis. IFN‐γ‐specific CD8^+^ T cells inhibit *in‐vitro* transformation of EBV‐infected B cells 47, 48. The cytokine exerts its effects via the canonical JAK–STAT pathway that is known to be over‐expressed in HRS cells 49, and therefore the IFN‐γ‐specific CD8^+^ T cell response is likely to be important in the pathogenesis of EBV^+^cHL. Thus IFN‐γ production was chosen as a highly relevant *in‐vitro* measure of effector function. Strikingly, approximately 90% of all HLA class I‐restricted LMP2A‐specific IFN‐γ‐producing CD8^+^ T cell responses generated were elicited through HLA‐A*02 compared to the A*01, A*03 and B*08 allotypes. Previous work using these cell lines assessed the response to endogenously processed EBV latency‐III antigens. In contrast, our novel approach enabled us to narrow our assessment to the EBV^+^cHL‐relevant protein LMP2A 25. From our *ex‐vivo* analysis (and consistent with previous work in healthy EBV‐seropositive individuals) 12, LMP2A‐specific IFN‐γ^+^ effector CD8^+^ T cell responses were modest, but none the less higher than for LMP1 or EBNA1. These data implicate LMP2A as a critical target for effector CD8^+^ T cells in the immunopathogenesis of EBV^+^cHL. This is consistent with the correlation between LMP2A, but not LMP1 or EBNA1, and intratumoral CD8 expression, with data showing that LMP1 and EBNA1 encode for peptides that stimulate IL‐10‐secreting regulatory T cells 38, 39, and with the observation that EBNA1 predominantly (but not exclusively) generates a CD4^+^ effector T cell response 12.

To substantiate the emerging hypothesis, we tested the influence of HLA class I on the hierarchy of CD8^+^ T cell responses across the spectrum of EBV latency antigens in healthy seropositive donors. The immunodominant (EBNA3A/3B/3C) and subdominant (LMP1/LMP2A) viral antigens were compared. To enable a comprehensive evaluation, in addition to functionally defined epitopes, we adopted computational analysis to predict novel HLA class I‐restricted epitopes. Predicted epitopes were selected using stringent criteria. The prerequisite for a predicted peptide to be a potentially immunogenic CD8^+^ T cell epitope is an ability to bind to an HLA class I molecule (although the magnitude of binding is not in itself an accurate forecast of immunogenicity) 54. Extensive assays were used to confirm HLA class I binding in all cases, with the exception of HLA‐B*37‐restricted LMP1/LMP2A peptides for which validated reagents were lacking. Equal numbers of defined and predicted peptides with equivalent HLA class I coverage for the immunodominant and subdominant EBV latency proteins were tested in a large number of healthy EBV‐seropositive donors. Strikingly, EBNA3A/3B/3C‐specific CD8^+^ T cell responses were stimulated by peptides presented by numerous HLA class I allotypes. By contrast, the relatively modest EBV latency‐II‐specific CD8^+^ T cell responses were confined largely to HLA‐A*02.

These findings indicate that HLA class I impacts EBV latency‐II antigen‐specific CD8^+^ T cell response hierarchies and provide a mechanistic basis for the immunopathogenesis of EBV^+^cHL. In EBV^+^ HRS cells, antigen presentation is intact and restoration of relevant EBV‐specific T cell immunity can induce EBV^+^cHL regression 8, 9, 10, 11. Indeed, HLA class I expression is more evident in EBV^+^cHL than EBV^–^cHL 7, 16. The lack of selection pressure to down‐regulate HLA class I in virus‐associated cHL indicates that this strategy is not required to evade tumour‐associated CD8^+^ T cell immunity. These results provide a mechanism by which HRS cells can avoid CD8^+^ T cell‐mediated immune attack despite intact antigen presentation.

The factors that determine CD8^+^ T cell immunodominance have largely been investigated in animal models and include antigen abundance, temporal antigen presentation, CD8^+^ T cell precursor frequency, the priming environment and T cell receptor (TCR)/peptide‐HLA class I binding affinity 55. Here, we demonstrate that HLA class I restrictions influence established EBV latency antigen‐specific CD8^+^ T cell hierarchies. One explanation for this observation might be preferential handling of EBV latency‐II antigens by the HLA‐A*02 processing pathway. Notably, for human cytomegalovirus, strong viral epitope‐specific responses restricted by HLA‐A*02 and HLA‐A*01 have been identified 56, suggesting that the influence of HLA class I on CD8^+^ T cell immunodominance may be virus‐dependent. Further studies are required to address the issue.

A limitation of this study is that the findings do not discriminate between reduced immune reactivity being due to diminished antigen recognition of non‐HLA‐A*02 latency‐II antigens, or alternatively an anergic phenotype or a reduced frequency of memory T cell precursors. After acute EBV infection, a large CD8^+^ T cell response is elicited which contracts subsequently into a smaller memory pool 57, 58, 59. Interestingly, it is known that at least one HLA‐A*02‐restricted lytic epitope reactivity present in the acute phase consistently disappears once infection resolves 57. Although this phenomenon has not been observed for latent epitope reactivity, we cannot rule out definitively that non‐HLA‐A*02 latency‐II antigens induced a T cell response in the acute phase, which was subsequently extinguished in the memory phase. Alternatively, non‐HLA‐A*02 latency‐II antigen‐specific CD8^+^ T cells may be relatively enriched in inhibitory receptors such as programmed cell death protein 1 (PD‐1), relative to HLA‐A*02 latency‐II antigen‐specific CD8^+^ T cells. Although there are major histocompatibility complex (MHC) class I multimers for HLA‐A*02 latency‐II antigen‐specific CD8^+^ T cells, the lack of defined epitopes means there are no such reagents to directly visualize and phenotype non‐HLA‐A*02 latency‐II antigen‐specific CD8^+^ T cells.

Population‐based studies have demonstrated a positive association between infectious mononucleosis and cHL 60. However, this does not necessarily implicate EBV in the aetiology of cHL. It may, instead, reflect a predisposition to a particular clinical response to primary EBV infection, perhaps as a consequence of a predating immune impairment. In addition, transformation of EBV latency‐II‐expressing benign B cells may represent an initiating event in the pathogenesis of EBV^+^cHL. EBV‐infected germinal centre B cells have been shown to express the EBV latency‐II pattern 61, 62, identical to that seen in the HRS cells of EBV^+^cHL, which are known to have an atypical germinal centre derivation 63. In this situation, differential EBV latency‐II‐specific effector CD8^+^ T cell immune surveillance might contribute to the pathogenesis of EBV^+^cHL by attenuating immune‐mediated destruction of premalignant B cells. Using functional assays that removed the confounding influence of co‐expressed HLA alleles, we confirmed that HLA class I status indeed conferred differential levels of EBV latency‐II‐specific effector CD8^+^ T cell immunity in healthy seropositive participants. Here, those peptide pools (4–6) that contained HLA‐A*02 restricted defined LMP2A epitopes contributed > 80% of the HLA‐A*02 CD8^+^ T cell response, whereas the remaining pools (1–3) provided < 20%. However, reduced *ex‐vivo* HLA‐A*02^–^ global (i.e. mediated through all co‐expressed HLA‐alleles) LMP1/LMP2A‐specific effector CD8^+^ T cell responses were observed only in EBV^+^cHL patients. These results suggest that EBV latency‐II‐specific effector CD8^+^ T cell immune surveillance is particularly relevant to the pathogenesis of EBV^+^cHL. Further investigations into associations with disease outcome may be informative; however, given the high response rate in cHL very large numbers of uniformly treated patients would be required to be sufficiently powered.

Collectively, our data provide new insights into the immunopathogenesis of EBV^+^cHL and suggest that even modest CD8^+^ T cell responses directed against tumour‐associated viral proteins may reduce the incidence of malignant disease. Further studies are required to determine if similar mechanisms are applicable to other malignancies with EBV latency‐II expression patterns, such as extranodal natural killer (NK)/T cell lymphoma and undifferentiated nasopharyngeal carcinoma 64, 65.

## Disclosure

The authors declare no disclosures.

## Supporting information

Additional Supporting information may be found in the online version of this article at the publisher's web‐site:

**Fig. S1.** Flow cytometry analysis gating strategy.

**Fig. S2.** *Ex‐vivo* Epstein–Barr virus (EBV)‐specific CD8^+^ T cell responses in healthy controls and comparison between responses against EBV latency‐II proteins.

**Fig. S3.** *In‐vitro* latent membrane protein (LMP) 2A‐specific CD8^+^ T cell cytotoxicity compared between HLA‐A*02 and non‐human leucocyte antigen (HLA)‐A*02 healthy control participants.

**Table S1.** Previously published human leucocyte antigen (HLA) class I associations with Epstein–Barr virus (EBV)^+^ classical Hodgkin lymphoma (cHL).
**Table S2.** Latent membrane protein (LMP)2A overlapping peptide pools.
**Table S3.** Predicted and defined peptide epitopes from Epstein–Barr virus (EBV) latent proteins.
