# Supplementary material for: The impact of HLA class I and EBV latency‐II antigen‐specific CD8+ T cells on the pathogenesis of EBV+ Hodgkin lymphoma
Source: Clin Exp Immunol. 2015 Nov 13;183(2):206–20. doi: 10.1111/cei.12716 (PMC4711160; doi:10.1111/cei.12716)
Supplement: Supplementary file 4 — Table S1. Previously published human leucocyte antigen (HLA) class I associations with Epstein–Barr virus (EBV)+ classical Hodgkin lymphoma (cHL). Table S2. Latent membrane protein (LMP)2A overlapping peptide pools. Table S3. Predicted and defined peptide epitopes from Epstein–Barr virus (EBV) latent proteins. [file CEI-183-206-s004.docx]

**Supporting Information:**

**Table S1.** Previously published HLA-class I associations with EBV^+^cHL.

| Number of Patients | | Susceptibility | Reference |
| --- | --- | --- | --- |
| HLA-A*01 | |  |  |
|  | 934 (278 EBV+) | Increased incidence of EBV^+^cHL | [1] |
|  | 516 (192 EBV+) | Increased incidence of EBV^+^cHL | [2] |
|  | 338 (78 EBV+ of 311 tested) | Increased incidence of EBV^+^cHL | [3] |
| HLA-A*02 | |  |  |
|  | 934 (278 EBV+) | Decreased incidence of EBV^+^cHL | [1] |
|  | 516 (192 EBV+) | Decreased incidence of EBV^+^cHL | [2] |
|  | 338 (78 EBV+ of 311 tested) | Decreased incidence of EBV^+^cHL | [3] |
| HLA-B*37 | |  |  |
|  | 338 (78 EBV+ of 311 tested) | Increased incidence of EBV^+^cHL | [3] |

**Table S2.** LMP2A overlapping peptide pools.

Italics denotes a predicted epitope, bold a defined epitope.

| Pool #1 |  | Pool #2 |  | Pool #3 |
| --- | --- | --- | --- | --- |
| **MGSLEMVPM**GAGPPSPG |  | GTQDQSLYLGLQHDGND |  | *LLLAAVASSY*AAAQRKL |
| PMGAGPPSPGGDPDGYD |  | YLGLQHDGNDGLPPPPY |  | SSYAAAQRKLLTPVTVL |
| SPGGDPD*GYDGGNNSQY* |  | GNDGLPPPPYSPRDDSS |  | RKLLTPVTVLTAVVTFF |
| *GYDGGNNSQY*PSASGSS |  | PPYS*PRDDSSQHIY*EEA |  | TVLTAVVTFFAICLTWR |
| SQYPSASGSSGNTPTPP |  | DSSQHIYEEAGRGSMNP |  | TFFAICLTWRIEDPPFN |
| GSSGNTPTPPNDEERES |  | EEAGRGSMNPVCLPVIV |  | TWR**IEDPPFNSL**LFALL |
| TPPNDEERESNEEPPPP |  | MNPVCLPVIVAPYLFWL |  | PFNSLLFALLAAAGGLQ |
| RESNEEPPPPYEDPYWG |  | VIVA**PYLFWLAAI**AASC |  | ALLAAAGGLQGIYVLVM |
| PPPYEDPYWGNGDRHSD |  | FWLAAIAASCFTASVST |  | GLQGIYVLVMLVLLILA |
| YWGNGDRHSDYQPLGTQ |  | ASCFTASVSTVVTATGL |  | LVMLVLLILAYRRRWRR |
| HSDYQPLGTQDQSLYLG |  | VSTVVTATGLALSLLLL |  | ILAYRRRWRRLTVCGGI |
|  |  |  |  |  |
| Pool #4 |  | Pool #5 |  | Pool #6 |
| WRRLTVCGGIMFLACVL |  | LGTLNLTTMFLLMLLWT |  | VAGILFILAILTEWGSG |
| GGIMFLACVLVLIVDAV |  | TMFLLM**LLWTLVVL**L**I**C |  | LAI*LTEWGSGNRTY*GPV |
| DAVL*QLSPLLGAVT*VVS |  | LWTLVVLLICSSCSSCP |  | GSGNR**TYGPVFMCL**GGL |
| PLLGAVTVVSMTLLLLA |  | LIC**SSCSSCPLSKILL**A |  | GPVFM**CLGGLLTMV**AGA |
| VVSMTLLLLAFVLWLSS |  | S**CPLSKILL**ARLFLYAL |  | GGLLTMVAGAVWLTVMS |
| LLAFVLWLSSPGGLGTL |  | LLARL**FLYALALLL**LAS |  | AGAVWLTVMSNTLLSAW |
| LSSPG**GLGTLGAAL**LTL |  | LAS*ALIAGGSIL*QTNFK |  | VMSNT**LLSAWILTA**GFL |
| GTLGAALLTLAAALALL |  | GGSILQTNFKSLSSTEF |  | SAWI**LTAGFLIFL**IGFA |
| LTLAAALALLASLILGT |  | NFKSLSSTEFIPNLFCM |  | GFLIFLIGFALFGVIRC |
| ALLASLILGTLNLTTMF |  | TEFIPNLFCMLLLIVAG |  | CYYCLTL*ESEERPPTPY* |
|  |  | FCMLLLIVAGILFILAI |  | *ESEERPPTPY*RNTV |

**Table S3.** Predicted and defined peptide epitopes from EBV-latent proteins.

|  | **Epitope** | **Protein** | **Score** | **Source** | **Binding/ Reference** |  | **Epitope** | **Protein** | **Score** | **Source** | **Binding/ Reference** |
| --- | --- | --- | --- | --- | --- | --- | --- | --- | --- | --- | --- |
| HLA-A*01 | |  |  |  |  | HLA-A*11 | |  |  |  |  |
|  | VLEKARGSTY | EBNA3A | 25 | Predicted | Confirmed |  | SSCSSCPLSK | LMP2A | 29 | Defined | [4] |
|  | TNEEIDLAY | EBNA3B | 26 | Predicted | Confirmed | HLA-A*24 | |  |  |  |  |
|  | CDEGTRHATTY | EBNA3B | 23 | Predicted | Confirmed |  | TYGPVFMCL | LMP2A | 24 | Defined | [5] |
|  | PKDAKQTDY | EBNA3C | 25 | Predicted | Confirmed |  | PYLFWLAAI | LMP2A | 22 | Defined | [6] |
|  | ASERLVPEESY | EBNA3C | 27 | Predicted | Confirmed | HLA-B*07 | |  |  |  |  |
|  | ELESSDDELPY | EBNA3C | 27 | Predicted | Confirmed |  | RPPIFIRRL | EBNA3A | 21 | Defined | [7] |
|  | YQEPPAPQAPY | EBNA3C | 25 | Predicted | Confirmed |  | QPRAPIRPI | EBNA3C | 23 | Defined | [7] |
|  | YQEPPPPQAPY | EBNA3C | 25 | Predicted | Confirmed |  | VPAPAGPIV | EBNA3A | 20 | Defined | [8] |
|  | LLALLFWLY | LMP1 | 21 | Predicted | Confirmed | HLA-B*08 | |  |  |  |  |
|  | WTGGALLVLY | LMP1 | 24 | Predicted | Confirmed |  | CPLSKILL | LMP2A | 26 | Defined | [9] |
|  | ESEERPPTPY | LMP2A | 27 | Predicted | Confirmed |  | QAKWRLQTL | EBNA3A | 32 | Defined | [10] |
|  | GYDGGNNSQY | LMP2A | 25 | Predicted | Confirmed |  | FLRGRAYGL | EBNA3A | 31 | Defined | [10] |
|  | PRDDSSQHIY | LMP2A | 25 | Predicted | Confirmed | HLA-B*35 | |  |  |  |  |
|  | LTEWGSGNRTY | LMP2A | 29 | Predicted | Confirmed |  | MGSLEMVPM | LMP2A | 8 | Defined | [11] |
| HLA-A*03 | |  |  |  |  |  | YPLHEQHGM | EBNA3A | 17 | Defined | [10] |
|  | ALFLGIVLF | LMP1 | 24 | Predicted | Confirmed |  | AVLLHEESM | EBNA3B | 7 | Defined | [8] |
|  | MLWRLGATI | LMP1 | 23 | Predicted | Confirmed | HLA-B*37 | |  |  |  |  |
|  | QLTEEVENK | LMP1 | 23 | Predicted | Confirmed |  | VDLLWLLLF | LMP1 | 28 | Predicted | Unconfirmed |
|  | ALIAGGSIL | LMP2A | 26 | Predicted | Confirmed |  | DEHHHDDSL | LMP1 | 25 | Predicted | Unconfirmed |
|  | LLLAAVASSY | LMP2A | 28 | Predicted | Confirmed |  | TEFIPNLF | LMP2A | 24 | Predicted | Unconfirmed |
|  | QLSPLLGAVT | LMP2A | 25 | Predicted | Confirmed |  | TDLSYIKSF | EBNA3A | 27 | Predicted | Unconfirmed |
|  | RLLLMRAGK | EBNA3A | 31 | Predicted | Confirmed |  | SDLRPLGSL | EBNA3B | 28 | Predicted | Unconfirmed |
|  | RVVVSAVVH | EBNA3A | 29 | Predicted | Confirmed |  | LDTQHILCF | EBNA3C | 28 | Predicted | Unconfirmed |
|  | IVSRGGPKVK | EBNA3A | 30 | Predicted | Confirmed |  | PDAPLDLSL | EBNA3C | 27 | Predicted | Unconfirmed |
|  | HLEPAQKGTK | EBNA3A | 28 | Predicted | Confirmed |  | LDFVRFMGV | EBNA3C | 17 | Defined | [12] |
|  | RLRAEAQVK | EBNA3A | 36 | Defined | [13] | HLA-B*44 | |  |  |  |  |
| HLA-A*02 | |  |  |  |  |  | VEITPYKPTW | EBNA3B | 25 | Defined | [8] |
|  | LLWTLVVLL | LMP2A | 30 | Defined | [4] |  | EGGVGWRHW | EBNA3C | 13 | Defined | [14] |
|  | FLYALALLL | LMP2A | 24 | Defined | [15] |  | EENLLDFVRF | EBNA3C | 25 | Defined | [16] |
|  | CLGGLLTMV | LMP2A | 27 | Defined | [17] |  | KEHVIQNAF | EBNA3C | 25 | Defined | [18] |
|  | LTAGFLIFL | LMP2A | 25 | Defined | [5] | HLA-B*60 (HLA-B*40.01) | | |  |  |  |
|  | YLLEMLWRL | LMP1 | 30 | Defined | [19] |  | IEDPPFNSL | LMP2A | 22 | Defined | [5] |
|  | YLQQNWWTL | LMP1 | 24 | Defined | [19] |  |  |  |  |  |  |
|  | TLLVDLLWL | LMP1 | 27 | Defined | [19] |  |  |  |  |  |  |
|  | LLLIALWNL | LMP1 | 28 | Defined | [19] |  |  |  |  |  |  |
|  | ALLVLYSFA | LMP1 | 19 | Defined | [20] |  |  |  |  |  |  |
|  | SVRDRLARL | EBNA3A | 23 | Defined | [10] |  |  |  |  |  |  |
|  | LLDFVRFMGV | EBNA3C | 22 | Defined | [21] |  |  |  |  |  |  |

**Figure S1.** Flow Cytometry analysis gating strategy. Gating on patient sample with positive control stimulation. Arrows indicate order from parent gate. Gating strategy follows previously published standard practice [22].

**Figure S2.** *Ex vivo* EBV-specific CD8^+^ T-cell responses in healthy controls and comparison between responses against EBV-latency-II proteins. Error bars represent SEM. **(A)** Summed percentages of *ex vivo* LMP1/2A-specific CD8^+^ T-cell responses defined by IFNγ, TNFα and CD107a in healthy EBV-seropositive controls. **(B-C)** Comparison of EBV-latency-II antigen-specific CD8^+^ T-cells. Summed percentages of IFNγ, TNFα and CD107a responses in **(B)** cHL patients and **(C)** healthy EBV-seropositive controls.

**Fig. S3.** *In vitro* LMP2A-specific CD8^+^ T-cell cytotoxicity compared between HLA-A*02^+^ and HLA-A*02^-ve^ healthy control donors. EBV-specific T-cells were expanded in four EBV-seropositive healthy control donors (Donors 1-4, HLA-A*02/A*02/B*07/B*44; HLA-A*03/A*23/B*35/B*44; HLA-A*02/A*02/B*35/B*57; HLA-A*03/A*32/B*8/B*47 respectively). **(A)** LMP2A-specific cytotoxicity was quantified using autologous CFSE labeled PHA blasts (target cells) incubated with each of six LMP2A overlapping peptide pools (Table S2). LMP2A-specific lysis was calculated for each well relative to the unpulsed control sample. **(B)** Representative intracellular cytokine and CD107ab mobilization by CD8^+^ T-cells (gated on CD3^+^) in response to 6 hours stimulation by LMP2A peptide pool #1 pulsed PHA blasts in donor 3 (top panel) compared with unpulsed PHA blasts (bottom panel).

**Supporting Information References**

1. Hjalgrim H, Rostgaard K, Johnson PC, Lake A, Shield L, Little AM, Ekstrom-Smedby K, Adami HO, Glimelius B, Hamilton-Dutoit S, Kane E, Taylor GM, McConnachie A, Ryder LP, Sundstrom C, Andersen PS, Chang ET, Alexander FE, Melbye M, Jarrett RF. HLA-A alleles and infectious mononucleosis suggest a critical role for cytotoxic T-cell response in EBV-related Hodgkin lymphoma*.* Proc Natl Acad Sci U S A 2010; **107**:6400-5.

2. Niens M, Jarrett RF, Hepkema B, Nolte IM, Diepstra A, Platteel M, Kouprie N, Delury CP, Gallagher A, Visser L, Poppema S, te Meerman GJ, van den Berg A. HLA-A*02 is associated with a reduced risk and HLA-A*01 with an increased risk of developing EBV+ Hodgkin lymphoma*.* Blood 2007; **110**:3310-5.

3. Huang X, Kushekhar K, Nolte I, Kooistra W, Visser L, Bouwman I, Kouprie N, Veenstra R, van Imhoff G, Olver B, Houlston RS, Poppema S, Diepstra A, Hepkema B, van den Berg A. HLA associations in classical Hodgkin lymphoma: EBV status matters*.* PLoS One 2012; **7**:e39986.

4. Lee SP, Thomas WA, Blake NW, Rickinson AB. Transporter (TAP)-independent processing of a multiple membrane-spanning protein, the Epstein-Barr virus latent membrane protein 2*.* Eur J Immunol 1996; **26**:1875-83.

5. Lee SP, Tierney RJ, Thomas WA, Brooks JM, Rickinson AB. Conserved CTL epitopes within EBV latent membrane protein 2: a potential target for CTL-based tumor therapy*.* J Immunol 1997; **158**:3325-34.

6. Burrows SR, Elkington RA, Miles JJ, Green KJ, Walker S, Haryana SM, Moss DJ, Dunckley H, Burrows JM, Khanna R. Promiscuous CTL recognition of viral epitopes on multiple human leukocyte antigens: biological validation of the proposed HLA A24 supertype*.* J Immunol 2003; **171**:1407-12.

7. Hill A, Worth A, Elliott T, Rowland-Jones S, Brooks J, Rickinson A, McMichael A. Characterization of two Epstein-Barr virus epitopes restricted by HLA-B7*.* Eur J Immunol 1995; **25**:18-24.

8. Rickinson AB, Moss DJ. Human cytotoxic T lymphocyte responses to Epstein-Barr virus infection*.* Annu Rev Immunol 1997; **15**:405-31.

9. Tsang ML, Munz C. Cytolytic T lymphocytes from HLA-B8+ donors frequently recognize the Hodgkin's lymphoma associated latent membrane protein 2 of Epstein Barr virus*.* Herpesviridae 2011; **2**:4.

10. Burrows SR, Gardner J, Khanna R, Steward T, Moss DJ, Rodda S, Suhrbier A. Five new cytotoxic T cell epitopes identified within Epstein-Barr virus nuclear antigen 3*.* J Gen Virol 1994; **75 ( Pt 9)**:2489-93.

11. Straathof KC, Leen AM, Buza EL, Taylor G, Huls MH, Heslop HE, Rooney CM, Bollard CM. Characterization of latent membrane protein 2 specificity in CTL lines from patients with EBV-positive nasopharyngeal carcinoma and lymphoma*.* J Immunol 2005; **175**:4137-47.

12. Shi Y, Smith KD, Kurilla MG, Lutz CT. Cytotoxic CD8+ T cells recognize EBV antigen but poorly kill autologous EBV-infected B lymphoblasts: immunodominance is elicited by a peptide epitope that is presented at low levels in vitro*.* J Immunol 1997; **159**:1844-52.

13. Hill AB, Lee SP, Haurum JS, Murray N, Yao QY, Rowe M, Signoret N, Rickinson AB, McMichael AJ. Class I major histocompatibility complex-restricted cytotoxic T lymphocytes specific for Epstein-Barr virus (EBV)-transformed B lymphoblastoid cell lines against which they were raised*.* J Exp Med 1995; **181**:2221-8.

14. Morgan SM, Wilkinson GW, Floettmann E, Blake N, Rickinson AB. A recombinant adenovirus expressing an Epstein-Barr virus (EBV) target antigen can selectively reactivate rare components of EBV cytotoxic T-lymphocyte memory in vitro*.* J Virol 1996; **70**:2394-402.

15. Lautscham G, Haigh T, Mayrhofer S, Taylor G, Croom-Carter D, Leese A, Gadola S, Cerundolo V, Rickinson A, Blake N. Identification of a TAP-independent, immunoproteasome-dependent CD8+ T-cell epitope in Epstein-Barr virus latent membrane protein 2*.* J Virol 2003; **77**:2757-61.

16. Burrows SR, Misko IS, Sculley TB, Schmidt C, Moss DJ. An Epstein-Barr virus-specific cytotoxic T-cell epitope present on A- and B-type transformants*.* J Virol 1990; **64**:3974-6.

17. Lee SP, Thomas WA, Murray RJ, Khanim F, Kaur S, Young LS, Rowe M, Kurilla M, Rickinson AB. HLA A2.1-restricted cytotoxic T cells recognizing a range of Epstein-Barr virus isolates through a defined epitope in latent membrane protein LMP2*.* J Virol 1993; **67**:7428-35.

18. Khanna R, Burrows SR, Kurilla MG, Jacob CA, Misko IS, Sculley TB, Kieff E, Moss DJ. Localization of Epstein-Barr virus cytotoxic T cell epitopes using recombinant vaccinia: implications for vaccine development*.* J Exp Med 1992; **176**:169-76.

19. Khanna R, Burrows SR, Nicholls J, Poulsen LM. Identification of cytotoxic T cell epitopes within Epstein-Barr virus (EBV) oncogene latent membrane protein 1 (LMP1): evidence for HLA A2 supertype-restricted immune recognition of EBV-infected cells by LMP1-specific cytotoxic T lymphocytes*.* Eur J Immunol 1998; **28**:451-8.

20. Duraiswamy J, Burrows JM, Bharadwaj M, Burrows SR, Cooper L, Pimtanothai N, Khanna R. Ex vivo analysis of T-cell responses to Epstein-Barr virus-encoded oncogene latent membrane protein 1 reveals highly conserved epitope sequences in virus isolates from diverse geographic regions*.* J Virol 2003; **77**:7401-10.

21. Kerr BM, Kienzle N, Burrows JM, Cross S, Silins SL, Buck M, Benson EM, Coupar B, Moss DJ, Sculley TB. Identification of type B-specific and cross-reactive cytotoxic T-lymphocyte responses to Epstein-Barr virus*.* J Virol 1996; **70**:8858-64.

22. Mahnke YD, Roederer M. OMIP-001: Quality and phenotype of Ag-responsive human T-cells*.* Cytometry A 2010; **77**:819-20.
